# Supplementary material for: Perilla frutescens Seed Residue Extract Restores Gut Microbial Balance and Enhances Insulin Function in High-Fat Diet and Streptozotocin-Induced Diabetic Rats
Source: Int J Mol Sci. 2025 Aug 22;26(17):8176. doi: 10.3390/ijms26178176 (PMC12428114; doi:10.3390/ijms26178176)
Supplement: Supplementary file 1 [file ijms-26-08176-s001.zip › ijms-3806778-supplementary.pdf]

Figure S1. HOMA-IR score.

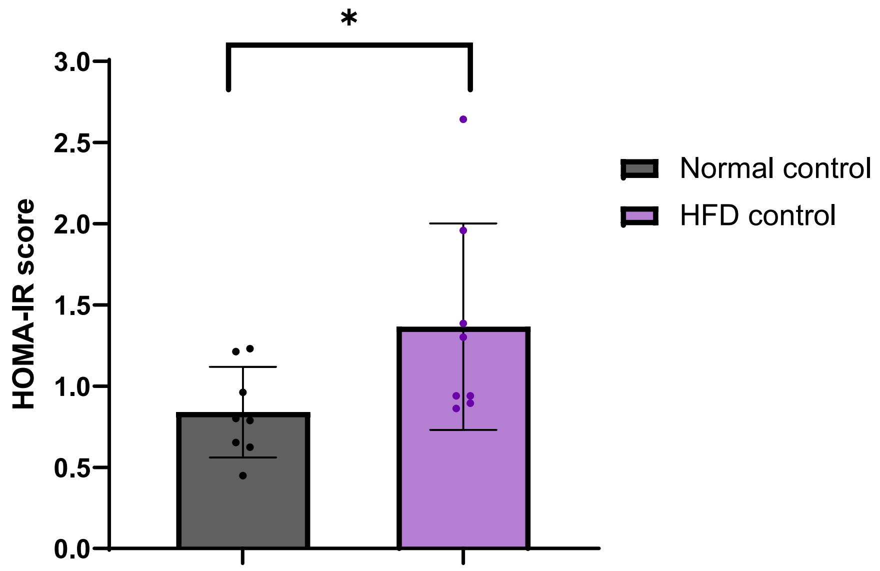

Figure S2. HPLC chromatogram (λ=320 nm) of PCE

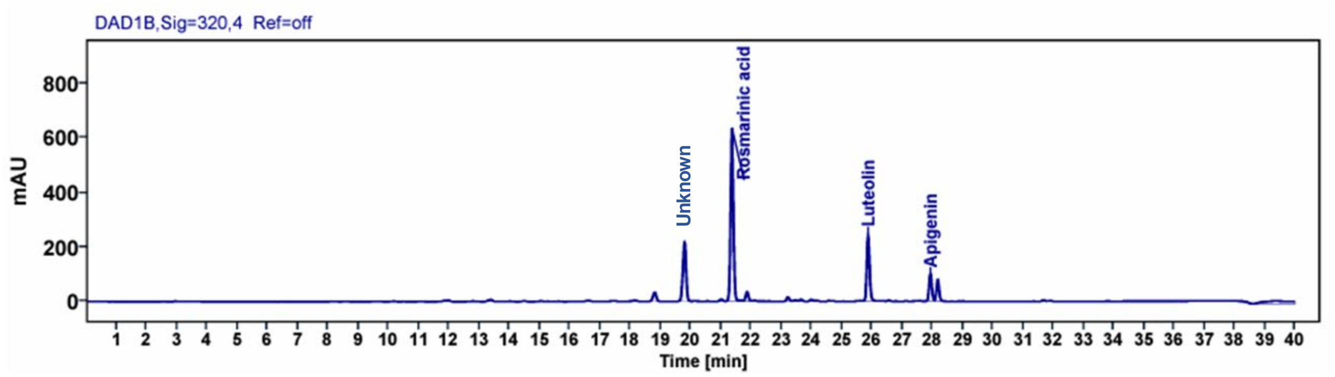

Figure S3. Alpha diversity at the genus level between normal control and HFD-STZ control group

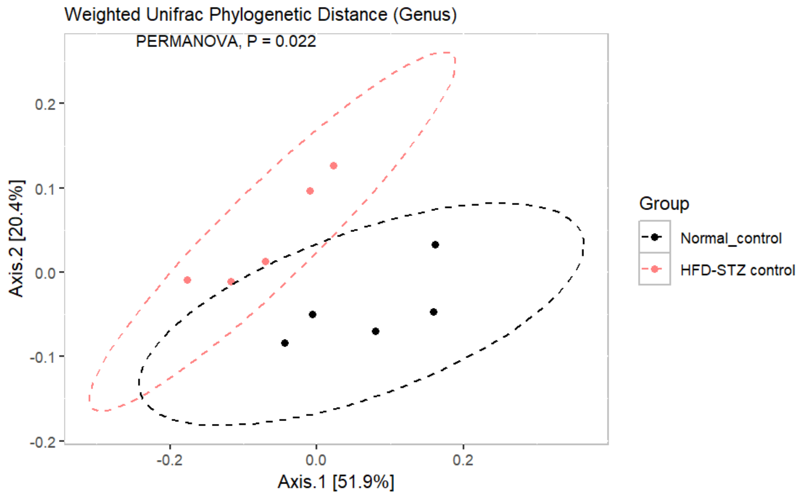

Figure S4. Heatmap correlation between key metabolic maker and gut microbiome.

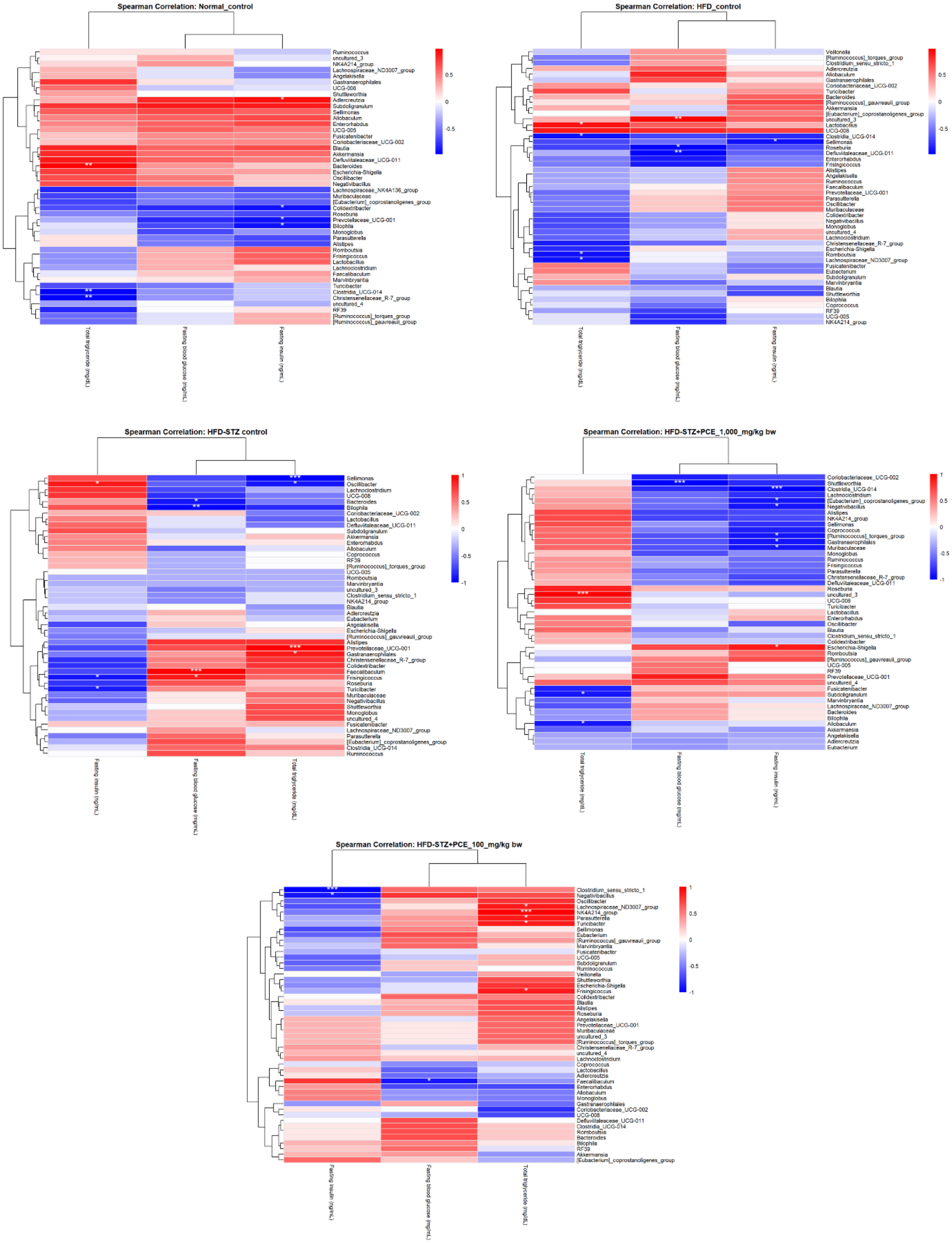

Table S1. The differential abundance bacteria in statistics for all detected taxa.

Name	Statistic			
	kw.ep	kw.eBH	glm.ep	glm.eBH
d__Bacteria.p__Actinobacteriota.c__Coriobacteriia.o__Coriobacteriales.f__Atopobiaceae.g__Coriobacteriaceae_UCG.002	0.001996	0.0617	1.11E-05	0.000243
d__Bacteria.p__Firmicutes.c__Clostridia.o__Lachnospirales.f__Lachnospiraceae.g__Fusicatenibacter	0.002454	0.0617	0.000169	0.001868
d__Bacteria.p__Bacteroidota.c__Bacteroidia.o__Bacteroidales.f__Prevotellaceae.g__Prevotellaceae_UCG.001	0.003087	0.0617	0.000497	0.003179
d__Bacteria.p__Firmicutes.c__Clostridia.o__Monoglobales.f__Monoglobaceae.g__Monoglobus	0.004591	0.0617	0.000161	0.001545
d__Bacteria.p__Firmicutes.c__Clostridia.o__Lachnospirales.f__Lachnospiraceae.g__Coprococcus	0.005208	0.0617	0.000352	0.00253
d__Bacteria.p__Firmicutes.c__Clostridia.o__Eubacteriales.f__Eubacteriaceae.g__Eubacterium	0.006324	0.063429	0.001435	0.006326
d__Bacteria.p__Firmicutes.c__Bacilli.o__Lactobacillales.f__Lactobacillaceae.g__Lactobacillus	0.006509	0.06209	0.00516	0.021184
d__Bacteria.p__Firmicutes.c__Clostridia.o__Lachnospirales.f__Lachnospiraceae.g__.Eubacterium._xylanophilum_group	0.00655	0.0617	0.006481	0.015088
d__Bacteria.p__Firmicutes.c__Clostridia.o__Oscillospirales.f__Ruminococcaceae.g__Subdoligranulum	0.006699	0.062105	0.001395	0.007639
d__Bacteria.p__Firmicutes.c__Incertae_Sedis.o__DTU014.f__DTU014.g__DTU014	0.007262	0.062205	0.002384	0.01102
d__Bacteria.p__Firmicutes.c__Clostridia.o__Christensenellales.f__Christensenellaceae.g__Christensenellaceae_R.7_group	0.007456	0.062149	0.005	0.019311
d__Bacteria.p__Firmicutes.c__Clostridia.o__Lachnospirales.f__Lachnospiraceae.g__Lachnospiraceae_NK4A136_group	0.007703	0.0617	0.000484	0.002596
d__Bacteria.p__Firmicutes.c__Bacilli.o__Erysipelotrichales.f__Erysipelatoclostridiaceae.g__Erysipelatoclostridium	0.008107	0.061847	0.003813	0.012809
d__Bacteria.p__Firmicutes.c__Clostridia.o__Lachnospirales.f__Lachnospiraceae.g__Shuttleworthia	0.008651	0.062105	9.26E-05	0.001039
d__Bacteria.p__Firmicutes.c__Clostridia.o__Oscillospirales.f__Oscillospiraceae.g__UCG.005	0.009911	0.063875	8.33E-05	0.001175

d__Bacteria.p__Actinobacteriota.c__Coriobacteriia.o__Coriobacteriales.f__Eggerthellaceae.g__Enterorhabdus	0.010032	0.064259	0.001485	0.007996
d__Bacteria.p__Firmicutes.c__Clostridia.o__Oscillospirales.f__Butyricicoccaceae.g__UCG.009	0.010441	0.063691	0.008989	0.031278
d__Bacteria.p__Bacteroidota.c__Bacteroidia.o__Bacteroidales.f__Prevotellaceae.g__Alloprevotella	0.011567	0.067383	0.001947	0.009751
d__Bacteria.p__Firmicutes.c__Clostridia.o__Lachnospirales.f__Lachnospiraceae.g__.Ruminococcus._gauvreauii_group	0.012738	0.068268	0.005126	0.019742
d__Bacteria.p__Firmicutes.c__Clostridia.o__Oscillospirales.f__UCG.010.g__UCG.010	0.014101	0.072681	0.008373	0.026508
d__Bacteria.p__Firmicutes.c__Bacilli.o__Erysipelotrichales.f__Erysipelotrichaceae.g__Faecalibaculum	0.014561	0.069553	0.001025	0.00646
d__Bacteria.p__Firmicutes.c__Clostridia.o__Oscillospirales.f__Ruminococcaceae.g__Ruminococcus	0.014708	0.071675	0.006421	0.025524
d__Bacteria.p__Firmicutes.c__Clostridia.o__Clostridiales.f__Clostridiaceae.g__Clostridium_sensu_stricto_1	0.014782	0.069858	0.001303	0.007001
d__Bacteria.p__Firmicutes.c__Bacilli.o__Erysipelotrichales.f__Erysipelatoclostridiaceae.g__Candidatus_Stoquefichus	0.014981	0.071836	0.001142	0.006835
d__Bacteria.p__Verrucomicrobiota.c__Verrucomicrobiae.o__Verrucomicrobiales.f__Akkermansiaceae.g__Akkermansia	0.017098	0.075723	0.025493	0.069019
d__Bacteria.p__Firmicutes.c__Clostridia.o__Oscillospirales.f__Ruminococcaceae.g__UBA1819	0.018367	0.077538	0.010759	0.033076
d__Bacteria.p__Firmicutes.c__Clostridia.o__Peptostreptococcales.Tissierellales.f__Anaerovoracaceae.g__.Eubacterium._nodatum_group	0.018857	0.079246	0.030617	0.073677
d__Bacteria.p__Firmicutes.c__Clostridia.o__Oscillospirales.f__Ruminococcaceae.g__DTU089	0.020569	0.079798	0.00197	0.008997
d__Bacteria.p__Firmicutes.c__Bacilli.o__Erysipelotrichales.f__Erysipelotrichaceae.g__Dubosiella	0.02317	0.088068	0.013317	0.026887
d__Bacteria.p__Firmicutes.c__Clostridia.o__Clostridia_UCG.014.f__Clostridia_UCG.014.g__Clostridia_UCG.014	0.024675	0.087168	0.029485	0.073561
d__Bacteria.p__Firmicutes.c__Clostridia.o__Lachnospirales.f__Lachnospiraceae.g__Marvinbryantia	0.025351	0.088341	0.006233	0.024861

d__Bacteria.p__Actinobacteriota.c__Coriobacteriia.o__Coriobacteriales.f__Eggerthellaceae.g__Gordonibacter	0.026585	0.091774	0.018891	0.046723
d__Bacteria.p__Firmicutes.c__Clostridia.o__Lachnospirales.f__Lachnospiraceae.g__.Eubacterium._ventriosum_group	0.026779	0.093833	0.013265	0.040445
d__Bacteria.p__Desulfobacterota.c__Desulfovibrionia.o__Desulfovibrionales.f__Desulfovibrionaceae.g__Bilophila	0.027018	0.094257	0.059673	0.125699
d__Bacteria.p__Firmicutes.c__Clostridia.o__Oscillospirales.f__Oscillospiraceae.g__Flavonifractor	0.027052	0.092553	0.032843	0.074566
d__Bacteria.p__Bacteroidota.c__Bacteroidia.o__Bacteroidales.f__Bacteroidaceae.g__Bacteroides	0.027595	0.09365	0.011706	0.039196
d__Bacteria.p__Firmicutes.c__Clostridia.o__Lachnospirales.f__Lachnospiraceae.g__CAG.56	0.027792	0.092517	0.015317	0.04346
d__Bacteria.p__Firmicutes.c__Clostridia.o__Peptostreptococcales.Tissierellales.f__Anaerovoracaceae.g__Family_XIII_AD3011_group	0.028226	0.09353	0.049223	0.103716
d__Bacteria.p__Firmicutes.c__Clostridia.o__Oscillospirales.f__Oscillospiraceae.g__Colidextribacter	0.030574	0.097248	0.035244	0.084133
d__Bacteria.p__Firmicutes.c__Clostridia.o__Oscillospirales.f__Oscillospiraceae.g__Oscillibacter	0.034483	0.105508	0.014709	0.046527
d__Bacteria.p__Firmicutes.c__Clostridia.o__Lachnospirales.f__Lachnospiraceae.g__Lachnospiraceae_FCS020_group	0.042016	0.112141	0.01122	0.032232
d__Bacteria.p__Firmicutes.c__Clostridia.o__Oscillospirales.f__Ruminococcaceae.g__Angelakisella	0.046998	0.123713	0.015526	0.04753
d__Bacteria.p__Firmicutes.c__Clostridia.o__Oscillospirales.f__Ruminococcaceae.g__.Eubacterium._siraeum_group	0.056922	0.140396	0.023396	0.054457
d__Bacteria.p__Firmicutes.c__Clostridia.o__Oscillospirales.f__Oscillospiraceae.g__Intestinimonas	0.061773	0.146873	0.031757	0.057127
d__Bacteria.p__Firmicutes.c__Clostridia.o__Oscillospirales.f__Ruminococcaceae.g__Pygmaiobacter	0.062248	0.143259	0.043613	0.075925
d__Bacteria.p__Firmicutes.c__Bacilli.o__RF39.f__RF39.g__RF39	0.067011	0.159051	0.038926	0.07908
d__Bacteria.p__Firmicutes.c__Clostridia.o__Oscillospirales.f__Oscillospiraceae.g__NK4A214_group	0.078883	0.174319	0.045868	0.104042

d__Bacteria.p__Firmicutes.c__Clostridia.o__Peptostreptococcales.Tissierellales.f__Peptostreptococcaceae.g__Intestinibacter	0.084469	0.176508	0.026012	0.058924
d__Bacteria.p__Firmicutes.c__Clostridia.o__Oscillospirales.f__Ruminococcaceae.g__Negativibacillus	0.103277	0.205973	0.047	0.10149
d__Bacteria.p__Firmicutes.c__Clostridia.o__Lachnospirales.f__Lachnospiraceae.g__Sellimonas	0.130812	0.249385	0.027651	0.072669
d__Bacteria.p__Firmicutes.c__Negativicutes.o__Veillonellales.Selenomonadales.f__Veillonellaceae.g__Veillonella	0.202108	0.307315	0.03406	0.071375

Table S2. High fat diet (50% fat, 15% protein, 30 % carbohydrate)

Ingredients	Amont (gram)
CP (normal diet)	320
Casein	240
Corn flour	20
Sucrose	80
Soybean oil	50
Lard	250
Margarine	100
Soybean meal	55
Mineral	13
Vitamin	13
